# Recent Advances of Mesoporous Silica as a Platform for Cancer Immunotherapy

**DOI:** 10.3390/bios12020109

**Published:** 2022-02-10

**Authors:** Albert Yu, Xiaoyong Dai, Zixian Wang, Huaqing Chen, Bing Guo, Laiqiang Huang

**Affiliations:** 1 Precision Medicine and Healthcare Research Center, Tsinghua-Berkeley Shenzhen Institute (TBSI), Shenzhen International Graduate School, Tsinghua University, Shenzhen 518055, China; yxz20@mails.tsinghua.edu.cn (A.Y.); dai-xy18@mails.tsinghua.edu.cn (X.D.); wzx21@mails.tsinghua.edu.cn (Z.W.); chq20@mails.tsinghua.edu.cn (H.C.); 2 Shenzhen Key Laboratory of Gene and Antibody Therapy, State Key Laboratory of Chemical Oncogenomics, State Key Laboratory of Health Sciences and Technology, Tsinghua University, Shenzhen 518055, China; 3 School of Science and Shenzhen Key Laboratory of Flexible Printed Electronics Technology, Harbin Institute of Technology, Shenzhen 518055, China; guobing2020@hit.edu.cn

**Keywords:** drug delivery, mesoporous silica nanoparticles, nanomaterial, cancer, immunotherapy

## Abstract

Immunotherapy is a promising modality of treatment for cancer. Immunotherapy is comprised of systemic and local treatments that induce an immune response, allowing the body to fight back against cancer. Systemic treatments such as cancer vaccines harness antigen presenting cells (APCs) to activate T cells with tumor-associated antigens. Small molecule inhibitors can be employed to inhibit immune checkpoints, disrupting tumor immunosuppression and immune evasion. Despite the current efficacy of immunotherapy, improvements to delivery can be made. Nanomaterials such as mesoporous silica can facilitate the advancement of immunotherapy. Mesoporous silica has high porosity, decent biocompatibility, and simple surface functionalization. Mesoporous silica can be utilized as a versatile carrier of various immunotherapeutic agents. This review gives an introduction on mesoporous silica as a nanomaterial, briefly covering synthesis and biocompatibility, and then an overview of the recent progress made in the application of mesoporous silica to cancer immunotherapy.

## 1. Introduction

Although there has been incredible development in the field of medicine from the late 20th century to the 21st century, cancer remains one of the toughest diseases to treat [1]. While traditional chemotherapy is a cornerstone of cancer treatment, it suffers from several key limitations: low aqueous solubility that leads to either low dosage or the use of toxic solvents [2], poor selectivity and specificity that leads to systemic cytotoxicity [3], and multidrug resistance that leads to low treatment efficacy and tumor recurrence [4]. Recent years have witnessed the development of new therapeutics such as targeted therapeutics and immunotherapeutics, which despite improving on some of the shortcomings, still have limitations similar to traditional chemotherapy.

Efforts have been made to overcome the limitations of cancer therapeutics. One promising direction is the use of nanoparticle-based drug delivery systems (DDS), which have many advantages. The use of nanoparticles can improve biodistribution, pharmacokinetics, and efficacy of therapeutic agents [5,6]. There are a variety of nanoparticles that have been employed as a nanocarrier, such as polymeric nanoparticles [7,8,9], dendrimers [10,11,12], liposomes [13,14,15], metallic nanoparticles [16,17], and other inorganic nanoparticles [18,19,20]. Recently, inorganic nanoparticles have garnered increased interest as a DDS, with mesoporous silica being a relatively new material for study and research. Mesoporous silica was first produced in 1992 by the Mobil Corporation, patented as Mobil Composition of Matter (MCM). In 1997, through an improved Stöber method of synthesis, ordered sub-micrometer mesoporous silica was prepared and termed MCM-41 [21]. The first reported use of MCM-41 as a DDS was in 2001 as a nanocarrier for ibuprofen [22]. Since then, the popularity of mesoporous silica nanoparticles (MSN) has steadily increased. MSN is characterized by high porosity, tunable pore size, high surface area, and simple surface functionalization [23].

Aside from utilization as a DDS, mesoporous silica has shown promise as a carrier for imaging modality. Imaging agents require chemical stability in physiological conditions, high signal-to-noise ratio (SNR), and long circulation time to be effective. Through encapsulation or functionalization of the imaging agents inside or on the surface of MSN, these criteria can be achieved. Mesoporous silica protects the imaging agents in vivo, allows for high SNR through high loading capacity, and lengthens circulation time. Thus, MSN can be used in MRI, PET, and CT modalities [24,25,26]. Therapeutics can also be co-loaded to achieve a theragnostic nanosystem to both monitor and treat.

Although a significant amount of nanocarriers show promising results at the laboratory level, only a surprisingly small percent of these pass through to clinical use. Difficulties of transitioning from laboratory to clinical use can be attributed in part to material toxicity and biocompatibility, efficacy of the formulation, and scalability of fabrication. Mesoporous silica scores well in these areas, being a relatively biocompatible material, at least on the macro scale. Material properties such as biocompatibility often change when scaling down to the micro scale and beyond. Further studies need to be made to come to a definite conclusion. MSN fabrication is simple, cost-effective, and easy to scale compared to other nanomaterials. Thus, MSN shows promise as a DDS as well as a carrier for imaging modality and other modalities of treatment.

Despite decades of research into these materials, only recently were MSN and similar materials investigated as a candidate to aid in immunotherapy [27]. Nanocarriers benefit immunotherapy in the same fashion as chemotherapy, namely through increased selectivity/targeting and improved biodistribution. The properties of MSN that make it a good nanocarrier for chemotherapeutics also apply to immunotherapy. Aside from acting as a carrier for immunotherapeutics, mesoporous silica as a material can act as an adjuvant with intrinsic or extrinsic immunomodulatory activity [28,29].

This review aims to provide a concise overview of the recent research progress and development of mesoporous silica as a nanomaterial and is organized as follows. First, it outlines the controlled synthesis of ordered mesoporous silica nanoparticles and other forms or counterparts such as hollow/rattle-type. Second, the review gives an overview of the interaction of mesoporous silica in the body. Third, a brief synopsis on cancer immunotherapy was introduced to facilitate the understanding of material-based immunotherapy. Then, recent progress in drug delivery, especially related to cancer immunotherapy using mesoporous silica and counterparts, is highlighted. Finally, the review concludes with a personal perspective on the direction of future research in this field.

## 2. Synthesis of Mesoporous Silica

It was in the early 1990s that the synthesis of mesoporous silica was first reported [30]. The original synthesis involved a week-long reaction based on sol-gel chemistry between a silica precursor and a surfactant. Surfactants were utilized as structure-directing agents (SDA) to obtain uniform pore size and ordered pore structure. Advancements in the field over the decades have resulted in great achievements in the synthesis of ordered MSN, with increased knowledge on sol-gel chemistry. The size and morphology of MSN as well as the size and structure of the pores can all be rationally designed and fine-tuned. This review focuses on the synthesis of mesoporous silica that is applicable to drug delivery and immunotherapy.

### 2.1. Ordered Mesoporous Silica Nanoparticles

**Figure 1:**
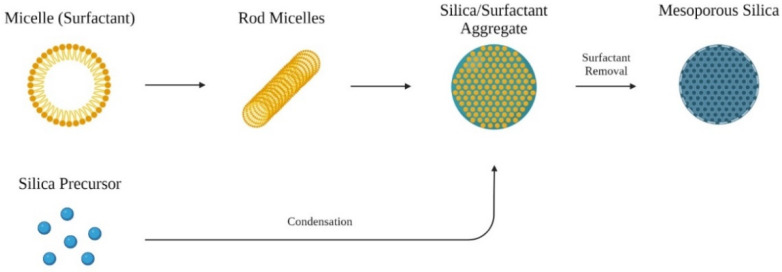
Formation of ordered mesoporous materials from surfactant and silica precursor.

For biomedical applications, MCM-41 has been extensively researched. The synthesis is relatively facile, with the surfactant cetyltrimethylammonium bromide (CTAB) as a liquid crystal templating agent, tetraethyl orthosilicate (TEOS) or tetramethyl orthosilicate (TMOS) as the silica precursor material [31], and alkali to act as a catalyst. In the basic aqueous solution, consisting of a mixture of water and alcohol with alkali, the silane hydrolyzes. Under alkaline conditions, the resulting silicate has negatively charged density and can assemble with the cationic surfactant CTAB through electrostatic interactions [32]. The surfactant CTAB would self-aggregate above the critical micelle concentration, resulting in silica condensing along the surface of the micelle. After further aggregation of multiple micelle-silica structures, removal of the surfactant results in MCM-41 type MSN (Figure 1).

For application in drug delivery and in the biomedical field in general, precision control over the properties of the mesoporous silica is necessary. The particle size and morphology of the resulting MSN can be controlled, ranging from tens of nanometers to a few hundred, based on the ratio of reactant concentrations [33]. The speed of agitation also contributes to the particle size, with higher stir speeds resulting in smaller particles. Pore size and pore orientation throughout the particle are dependent on the surfactants used as the SDA. For example, Pang and Tang showed that using different silica precursors and surfactants could change particle size. Using TEOS as a precursor resulted in a lower specific surface area and smaller pore size compared to using sodium silicate. Additionally, it was discovered that tuning the molar ratios of co-surfactants Triton X-100 and CTAB could change the morphology. A molar ratio of 0.6 Triton X-100: CTAB formed spherical morphology, but small deviations in the molar ratio caused the morphology to change to rod-like. Significantly increasing the molar ratio to 2.35 Triton X-100: CTAB resulted in amorphous materials with a lack of ordered structure [34].

MCM-48 is another member of the M41S mesoporous silica family of materials. Unlike the 2D hexagonal MCM-41 which has unidirectional channels, the 3D cubic mesostructure of MCM-48 has bicontinuous channels [35]. This slight difference in morphology influences the loading and release of molecules from the particle, which can facilitate inclusion and diffusion profiles of therapeutics. Early synthesis methods involved cationic-anionic surfactants, such as the SDA, with a time-consuming reaction performed at high temperatures [36]. However, the resulting particles are in the range of micrometers and thus are not suitable for use as nanocarriers. A modified Stöber method of synthesis was later discovered, using triblock copolymer pluronic F127 as a particle dispersion agent along with CTAB. This method resulted in monodispersed MCM-48 with particle size ranging from 150 to 600 nm, which is applicable to drug delivery [37].

Another extensively researched type of mesoporous silica that finds use in drug delivery is Santa Barbara Amorphous 15 (SBA-15). The 2D hexagonal-structured mesoporous silica was first synthesized in 1998 using a highly acidic medium with amphiphilic triblock copolymer P123 as the SDA. Compared to MCM-41, SBA-15 has larger pore size and thicker walls [38]. Sphere and rod-like SBA-15 in the micrometer scale, as well as other materials from the SBA family, have been synthesized and have found some biomedical applications. Table 1 summarizes some of the characteristics of different types of ordered MSN.

### 2.2. Hollow/Rattle-Type Mesoporous Silica Nanoparticles

Hollow/rattle-type MSN, as the name suggests, has a mesoporous silica shell and a hollow core. With low density and high specific area, this type of mesoporous silica is ideal for drug delivery systems with incredibly high loading capacity, capable of exceeding 1 g of drug per gram of silica [42]. Thus, lower amounts of these hollow mesoporous silica nanoparticles (HMSN) are required compared to the conventional MSN such as MCM-41. This can reduce the potential accumulation of silica in the body. However, there are issues with structural stability of weak shells after template removal which can result in the collapse of the shell during loading.

**Figure 2:**
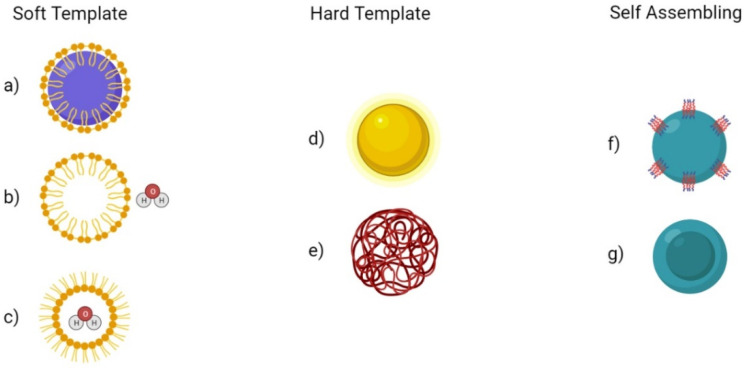
Fabrication methods for Hollow MSN (HMSN). (**a**) Dual surfactant soft template method (**b**) oil-in-water soft template method (**c**) water-in-oil soft template method (**d**) Metal and metal oxide hard template method (**e**) Polymer beads hard template method (**f**) Self-templating method of self-assembly (**g**) Selective-etching method of self-assembly.

HMSN can be fabricated using various methods, though conventionally it was through a dual template method. The dual template method involves one template being used to generate a hollow interior and another template as a pore forming agent to induce a mesoporous structure to the shell [43]. After the sol-gel process generates the mesoporous shell, the template could be removed through calcination or solvent extraction. For rattle-type particles, additional steps must be made to generate a silica core inside a mesoporous silica cage [44]. The fabrication process for these complex structures is difficult as well as costly and tedious. Newer methods have been investigated that allow for simpler and more scalable fabrication. Notable methods include soft templating, hard templating, and self-templating (Figure 2).

#### 2.2.1. Soft Templating

Synthesis using the soft template method relies on dual surfactants to simultaneously structure the hollow core and the mesoporous shell. For example, Li and colleagues utilized asymmetric triblock copolymer poly(styrene-b-2-vinyl pyridine-b-ethylene oxide) (PS-b-P2VP-b-PEO) and cationic CTAB [45]. The PS-b-P2VP-b-PEO acted as a stable template for the hollow core while CTAB acted as the template to form mesopores in the shell. Other methods for soft template fabrication of HMSN used oil-in-water [46] and water-in-oil [47] microemulsion. Mou and co-worker fabricated HMSN with the water-in-oil microemulsion method. A microemulsion was formed using cyclohexane, Triton X-100, and hexanol in water. TEOS and aminopropyltrimethoxy silane (APTMS) were used as silica precursors. These microemulsion methods generally produce particle size in the tens of nanometers. There are disadvantages to using soft templates as the fabrication method. The large amount of surfactants needed limits scalability. Some shortcomings include the dispersity of nanoparticles, as well as lack of control over particle size and mesoporous shell thickness.

#### 2.2.2. Hard Templating

Hard templating solves the issue of size distribution in the soft template method of fabrication, producing monodispersed and mesostructured HMSN. However, there are a few criteria that need to be met [48]. First, the silicification on the surface of the template must be faster than the self-condensation of silica in the bulk solution. This requires the surface to have functional groups that allow for interfacing with silicates under reaction conditions. Second, the stability of the template should be guaranteed. Leaching of the template into a solution due to favoring stronger interactions with the silica in solution can cause fabrications to fail. Third, the ease of removal of the template should be considered. Ease of fabrication on the surface of a template is rendered useless if the template cannot be removed without damaging the integrity of the mesoporous shell.

Polymer beads have been used as hard templating agents to great success due to materials such as polystyrene and polymethylmethacrylate. These polymer beads are inexpensive, uniformly sized, and removable by calcination, making them attractive for use as templating agents [49]. Usually, the surfaces of these polymers require activation by introducing certain functional groups to allow for surface silicification.

Metal and metal oxide nanoparticles can also be used as a hard template for MSN and can often remain in the MSN as part of the nanocarrier system. Metal and metal oxide nanoparticles such as Fe_3_O_4_ not only provide a template for mesoporous silica fabrication but also can assist in the delivery of the system to the target site or provide a modality of treatment such as photothermal or photodynamic therapy. Hollow MSN are possible by etching away the core with acid, though it is a complex and hazardous procedure. Aside from iron oxide, gold and platinum are other widely used templates [50,51,52].

#### 2.2.3. Self-Assembling

Self-templating HMSNs are produced through simple procedures without the need for additional templates and surfactants [53]. This method first involves the fabrication of template nanomaterials and then the formation of mesoporous silica from these templates. Ren et al. first introduced the construction of HMSN using cationic polyelectrolyte precoated mesoporous silica spheres with alkaline treatment as a template. The spheres were coated with poly-dimethyldiallylammonium chloride (PDDA) and treated with ammonia. The formation of anionic silicates and the subsequent electrostatic interactions with the coating formed a hollow silica shell [54]. With an appropriate selection of etching reagents and cores, HMSN can be tuned to a wide range of particle sizes [55,56].

Selective etching is another self-assembling method of HMSN fabrication. Utilizing both organosilane precursors and silica precursors, the method was developed based on the difference in properties between the two precursors. Namely, different stability under extreme temperature or pH conditions and different reactivity to etching agents. Through careful and elaborate design, encompassing both silica precursors into a template allows for the etching out of a hollow interior without disturbing the shell [57]. Surface protectors can be added to keep the mesoporous shell intact while the core is etched.

## 3. Interaction of Mesoporous Silica in the Body

**Figure 3:**
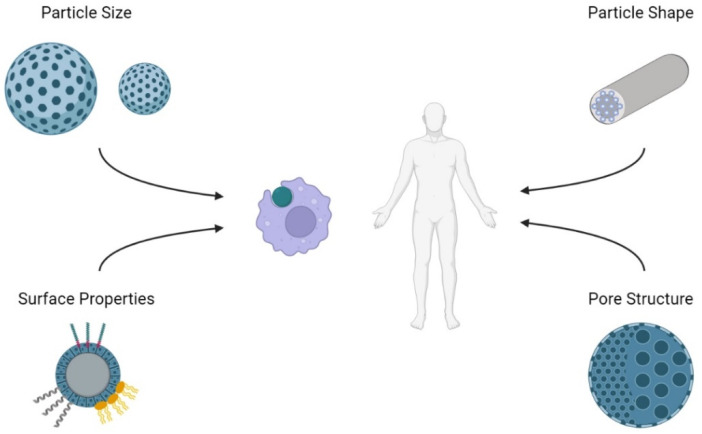
Properties of mesoporous silica and their effect in vivo. Effect of particle size, particle shape, surface functionalization, and pore structure should be considered in regard to cellular uptake, biodistribution, and toxicity.

With a unique mesoporous structure and high specific surface area, MSNs have a wide range of applications in biomedicine. For in vivo applications such as drug delivery and diagnostics, biocompatibility should be considered to avoid harm to the body. According to the consensus of the European Society for Biomaterials in 1986, “biocompatibility” was defined as “the ability of a material to perform with an appropriate host response in a specific application” [58]. Since then, nanomaterials and biomaterials have garnered significant interest and research. A reassessment of the biocompatibility of materials is necessary when transitioning to the micro- or nano-scale. Understanding how mesoporous silica affects the body can aid in designing and engineering more biocompatible MSNs (Figure 3).

### 3.1. Effect of Size

Particle size has a variety of effects on the interaction of the nanoparticle in vivo. A study by Mou and co-workers showed that cellular uptake by HeLa cells is size dependent [59]. From a selection of MSNs sized 30 nm, 50 nm, 110 nm, 170 nm, and 280 nm, the cellular uptake was maximized at a size of 50 nm. Though this can be a positive for drug delivery, the results could also suggest nonspecific biodistribution at small particle sizes. Another investigation performed by Shi and colleagues assessed biodistribution and urinary excretion of intravenously administered MSNs with sizes 80 nm, 120 nm, 200 nm, and 360 nm [60]. The smaller particles had longer blood-circulation lifetimes and larger particles had increased excretion through urine, suggesting a change in degradation rate due to a difference in the smaller and larger MSNs. Although size seems to be an obvious cause for the difference in degradation rate, some consensus is on particle porosity, which could increase with particle size [61]. Additionally, particles smaller than 10 nm risk renal clearance [62] while particles greater than 200 nm risk activating the complement system immune response [63]. Wang et al. investigated the cytotoxicity of MSN with particle size ranging from 30 nm to 200 nm on fibroblastic NIH3T3 cells [64]. MSNs of different concentrations ranging from 0 to 300 µg/mL were used for treatment on the cells. The larger particles had less cytotoxicity, with 200 nm sized MSNs showing the minimum cytotoxicity at concentrations no greater than 100 µg/mL. Hemolytic assays showed increased hemolytic activity at larger particle sizes, suggesting that a relatively safe MSN particle size range would be 100 to 200 nm [65,66].

### 3.2. Effect of Shape

There has been evidence suggesting that non-spherical particles have reduced phagocytosis by macrophages and longer in vivo circulation times [67]. Non-spherical particles have been shown to have better localization to blood vessel walls and subsequent extravasation, specifically ellipsoids, discoid shapes, and rods with high aspect ratios [68,69,70]. This is thought to be caused by the flow-induced rolling in shapes with high aspect ratios, resulting in edge margination. Though these results are related to nanoparticles in general, they can be applied to mesoporous silica as well. Rod-like MSN have been shown to have better cellular uptake, perhaps from a larger contact area with the cell membrane along the longitudinal axis [67]. One investigation showed that shape did not significantly affect toxicity, though high aspect ratio MSN had lower hemolytic activity [71].

### 3.3. Effect of Surface Functionalization

Surface properties of nanoparticles are important to consider when assessing biocompatibility. Nanoparticles that are positively charged would induce more immune response and cytotoxicity than neutral and negatively charged nanoparticles but benefit from more readily traversing mucosal barriers [63]. Exposed silanol groups on the mesoporous silica can interact with and damage molecules in vivo [72]. Pure, nonfunctionalized MSNs have negative zeta potential and would associate with serum opsonin immediately after intravenous injection. The opsonin associated MSN would then be cleared by the reticuloendothelial system (RES). Thus, surface modification is critical in improving circulation time and biocompatibility. For MSN, the process of functionalization is relatively facile.

A common functionalization is PEGylation, or the addition of polyethylene glycol (PEG). PEG can shield nanoparticles such as MSN from opsonization, acting as a stealth coating from the immune system. However, it is not an all-powerful functionalization as repeated exposure to PEGylated particles results in the production of anti-PEG antibodies that can rapidly remove subsequent doses [73]. Still, PEGylation has benefits such as lessening hemolytic activity and endocytosis from nonspecific cells [74]. PEGylation can also serve as a linker to add other functionalization.

With the abundance of silanol groups, surface modifications involving silanes with different functional groups can be achieved. Amino groups are a common addition with (3-aminopropyl)triethoxysilane (APTES) or (3-aminopropyl)trimethoxysilane (APTMS) being used. The silane end bonds with the silanol of the MSN and the amino end is free for functionalization. Lin and colleagues functionalized MSN with fluorescein isothiocyanate and folate using APTMS as a linker [75].

Lipid coating can also be utilized to improve biocompatibility. A PEGylated and phospholipid coated 200 nm MSN was shown to have superior suspensibility in PBS and significantly lower nonspecific binding in vitro [76]. Another publication reported the coating of a 65 nm MSN by a lipid membrane with PEG mixed in. Due to the modified lipid membrane, the resulting MSN had a high loading capacity of both hydrophilic and hydrophobic drugs while showing no major systemic cytotoxicity [77]. Another investigation involved a lipid-coated MSN providing sustained release and reducing premature leakage. The cationic liposome also interacted with cell membranes to improve cellular uptake [78].

Targeting molecules can be attached to MSN to add active targeting capabilities. Employing active targeting can allow for greater accumulation of nanoparticles to a specific site or specific cells. Monoclonal antibodies (mAbs) are one of the most prevalently used proteins for targeting. The antigen binding site of mAbs is highly selective for the cells of interest [79]. mAbs can be created whenever a new target protein is found on the cell surface [80]. However, there can be some constraints with size, with the average mAb having a molecular weight around ~150 kDa and a size around 10 nm [81]. A solution to this issue is to use only the essential binding domains of the antibodies [82]. These more compact targeting proteins are called antibody fragments (Fabs) and can allow for high ratios of targeting ligand: MSN. Aside from proteins, peptides can also act as a targeting modality. Peptides have the advantage of size over proteins but have limitations regarding structural stability and hydrophobicity [83,84]. A common peptide used is RGD, which targets upregulated integrins on tumor cells [85]. Another prevalent type of peptides is the cell-penetrating peptide or protein transduction domain (CPP/PTD) which acts on the cell membrane to enhance cellular uptake [86]. MSN can also utilize aptamers, which are short strands of RNA or DNA that can interact with biomarkers of tumor cells in a manner similar to the protein-receptor interaction [87]. Lastly, small molecules such as folate and hyaluronic acid that target overexpressed receptors can be utilized as a simple yet effective targeting method [88]. Table 2 summarizes these targeting modalities used with MSN and gives some examples.

### 3.4. Effect of Pore Structure

High porosity benefits drug delivery as a higher surface area allows for greater amounts of drug encapsulation. However, increased pore size can also pose the risks of increased reactivity and oxidative stress [61,105]. Nanoparticles with larger surface areas have more exposed silanol groups that can generate reactive oxygen species and damage the structure of biomolecules. However, other investigations suggest that the culprit of hemolytic activity [65] and cytotoxicity [106] is increased specific surface area, which is dependent on particle size.

## 4. Cancer Immunotherapy

Immunotherapy refers to the suppression or activation of the immune response with the goal of treating a disease. Immunosuppressive therapy aims to calm the immune system down and reduce inflammation. However, cancer growth is often tied to evasion from and suppression of the immune system. Cancer immunology thus aims to reactivate the immune system in targeting cancer cells.

Recent years have seen a surge of development surrounding the field of cancer immunotherapy. In 2011, ipilimumab, a monoclonal antibody (mAb), was approved by the FDA [107]. The therapeutic worked by targeting cytotoxic T-lymphocyte-associated protein 4 (CTLA-4), a receptor expressed on regulatory T cells (Tregs). CTLA-4 is essentially an immune checkpoint, or the “off” switch of the immune response [108]. Due to a blockade of CTLA-4 from the B7 ligand of antigen presenting cells (APCs) by ipilimumab, T cell activation is enabled. Since then, a flood of new immune-oncology agents have been researched and approved. Immunology can thus be classified based on the area of effect, whether systematic or local. Systematic immunology induces a full body immune activation for cancer and can include systemic cytokine treatment, cancer vaccines, and adoptive cell transfers (ACT) [109]. Local immunology ameliorates the immunosuppressive tumor microenvironment (TME) and encompasses monoclonal antibodies, other small molecular inhibitors, and drugs [110]. Immunotherapy has garnered interest and proven to be as effective as traditional cancer therapies [111].

**Figure 4:**
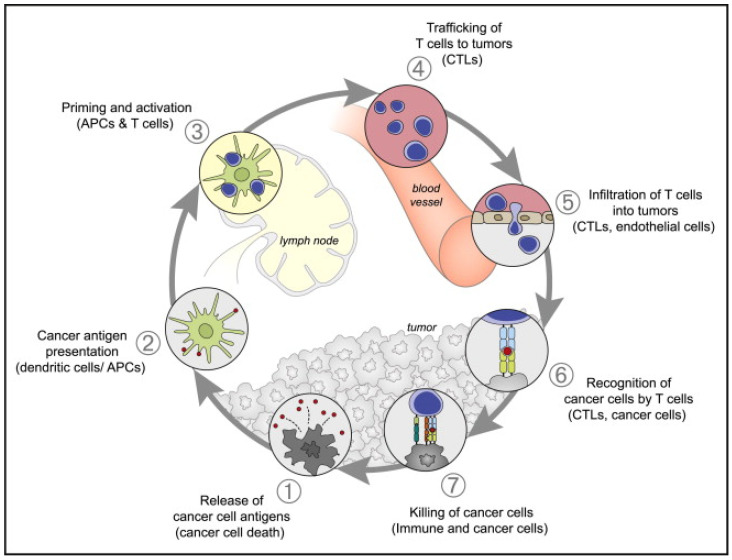
The Cancer-Immunity Cycle. The generation of an immune response to cancer is cyclical and can be divided into seven major steps. It starts from release of tumor-associated antigen and ends with the killing of cancer cells by T cells. Abbreviations: APCs, antigen presenting cells; CTLs, cytotoxic T lymphocytes. Reprinted with permission from Ref. [112]. Copyright 2021, Elsevier Inc.

Although elimination of cancer by cytotoxic T cells is the end goal of immunotherapy, there are numerous steps to take to ensure that the “cancer-immunity cycle” (Figure 4) continues [112]. Antigens are released from necrotic or apoptotic tumor cells and captured by APCs. The antigen is then presented on major histocompatibility complex (MHC) class I and II molecules. The APCs prime and activate effector T cells, and a subsequent response is based on the balance between T effector cells and T regulatory cells. Activated tumor-specific cytotoxic T lymphocytes (CTL) traffic to and infiltrate the TME. The CTL recognize and bind to cancer cells and ultimately kill them. Thus, this loops back to antigen release. Immunotherapy effectively starts and keeps this cycle running, either by providing antigens (cancer vaccine), CAR-T cells, or immunomodulatory molecules.

Application of nanomaterials to immunotherapy can greatly benefit the efficacy. Tumor antigen delivery for cancer vaccines suffers from inefficiencies in delivery [113,114] that can be solved by nanocarriers. Antigen vaccines, administered intramuscularly or subcutaneously, diffuse into the blood vessels and spread system wide but have limited delivery to secondary lymphoid organs. Delivery to these lymphoid organs is necessary to elicit a strong immune response. Nanocarriers for the tumor antigen can be functionalized to target the lymph nodes. As for immune checkpoint inhibitors, the lack of selectivity in the free drug form can lead to significant cytotoxicity related to the immune response [115,116,117]. Compared to traditional chemotherapy or even targeted therapeutics, a case can be made that immunotherapy is a more efficient and effective cancer treatment [118,119,120].

## 5. Immunotherapy and Mesoporous Silica

Mesoporous silica has many properties that make it a choice candidate for use in biomedicine, namely high surface area, large pore volume, and facile surface functionalization. MSN can improve co-delivery and distribution of antigens along with adjuvants or other therapeutics in high doses to critical sites such as the lymph nodes. A few recent applications of mesoporous silica as a platform for cancer immunotherapy are discussed below. Table 3 provides an overview of the content.

### 5.1. MSN as Self-Adjuvant

Adjuvants are substances that enhance the magnitude of immune response. Thus, they are often used in conjunction with antigens to improve immunogenicity and to allow for the formation of immunological memory for the antigens [107,143]. Mesoporous silica has a beneficial property of self-adjuvanticity, in which the pure material by itself provides adjuvant properties [144,145]. Mahony et al. investigated the self-adjuvant property of MSN with ovalbumin (OVA), a model protein antigen [146]. OVA + MSN was compared to free OVA + QuilA (a saponin-based adjuvant). Although the OVA + QuilA group had a stronger antibody response, the OVA + MSN group had 5 to 25-fold less antigens in the formulation. It can thus be concluded that MSN has adjuvant properties comparable to traditional adjuvants. The morphology of mesoporous silica can also play a role in adjuvanticity. Asymmetric head-tail mesoporous silica nanoparticles (HTMSN) induced a higher expression of CD40 and CD86 maturation markers on APCs compared to spherical MSN. This higher potential for stimulating APC maturation is presumed to be due to increased uptake of mesoporous silica with asymmetric morphology [147]. Biodegradable MSN frameworks also act as an aid in enhancing immunogenicity. Coupled with a tumor killing therapy such as PTT, tumor-associated antigens can be released and then be acquired by degraded MSN debris. These antigen-debris can then escape the necrotic tissue and selectively enter immune organs, stimulating immune response [121].

### 5.2. Cancer Vaccines

Cancer vaccines employ tumor-specific antigens to activate APCs and to elicit an immune response against the tumor. Mesoporous silica has emerged as a potential nanocarrier for the delivery of cancer vaccines. MSNs have the property of enhancing antitumor immunity through the possible dual loading of antigen and adjuvant on one platform, allowing for synchronized activity. Efforts have been made to improve the adjuvant properties of mesoporous silica.

Fontana et al. applied acetylated dextran, a biodegradable and biocompatible polymer, to mesoporous silica nanoparticles to aid in adjuvant properties [122]. Two nanovaccines were fabricated, one of which was MSNs coated with cancer cell membranes (CCMs) harvested from the solid tumors of the patient as a biomimetic. This coating conveyed the same antigens as that of the cancer cell. This opens new horizons in the field of personalized medicine, though it is limited to use with solid tumors and can be difficult to fabricate. The other MSN nanovaccine was functionalized with a model antigen Trp2 to evaluate the efficacy of the system as an adjuvant. The CCM-coated silica nanoparticles enhanced the secretion of interferon γ (IFN-γ) in peripheral blood mononuclear cells, polarizing primed T cells towards a Th1 cell-mediated response. The Trp2 functionalized silica nanoparticles showed high cytocompatibility with two human immortalized cell lines.

**Figure 5:**
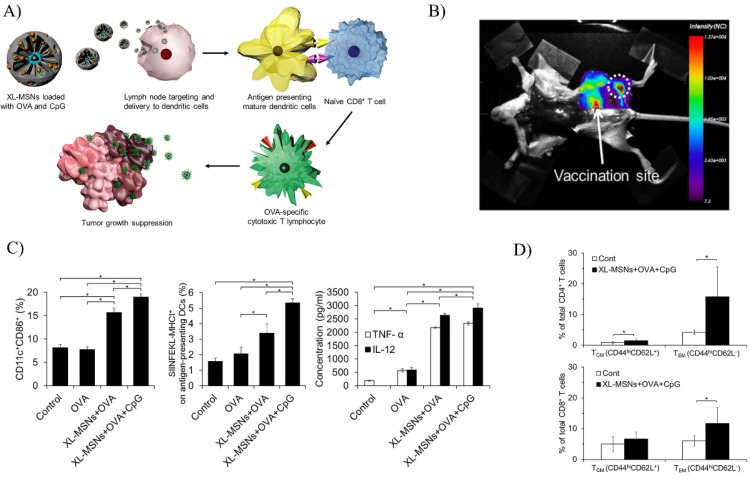
An extra-large pore MSN platform for co-delivery of antigen and agonist. (**A**) A schematic of the fabrication and vaccination process of the extra-large pore MSN (XL-MSN) co-loaded with antigen ovalbumin (OVA) and toll-like receptor 9 agonist CpG. (**B**) Fluorescence imaging of mouse injected with RITC-labeled XL-MSN. Shows accumulation of nanoparticles towards the lymph nodes. (**C**) Percentage of activated CD11c + CD86 + BMDCs and percentage of BMDCs presenting antigenic SIINFEKL peptide on the MHC-molecule. Analysis was through flow cytometry. Concentrations for secreted cytokines TNF-α and IL-12 from BMDCs measured by ELISA. (**D**) Memory T cell population for CD4+ and CD8+ T cells of vaccinated mice measured by flow cytometry. Error bars, mean ± s.d., * *p* < 0.05. Reprinted (adapted) with permission from Ref. [123]. Copyright 2021, American Chemical Society.

Cha and colleagues utilized extra-large pore MSNs to co-deliver OVA antigen and CpG oligonucleotide, a toll-like receptor 9 (TLR9) agonist (Figure 5) [123]. The MSNs were prepared with a particle size around 100 to 200 nm, regular pore size of 3 nm, and expanded pore size of 25 nm. The OVA was used as a model antigen and the unmethylated CpG acted as an agonist to TLR9 in dendritic cells to enhance expression of costimulatory molecule CD86. CD86 is required for priming of CTLs together with MHC I complex. In a test comparing free OVA and OVA-MSN, the OVA-MSN generated higher levels of APCs. Adding CpG to the MSN alongside OVA produced the highest level of APCs (Figure 5C). Accumulation of the MSN in lymph nodes was observed (Figure 5B). Immune memory was also induced to great effect, with significantly higher levels of memory T cells in the vaccinated mice compared control (Figure 5D).

Liu et al. fabricated a thin shell hollow mesoporous silica nanoparticle (THMSN) etched onto polyethyleneimine (PEI) to form a hybrid PEI-THMSN [124]. PEI was used as a nucleotide delivery agent, adding a positive charge to the MSN. As a result, the self-adjuvant properties of MSN were improved. To investigate this adjuvant effect, Trp2 was used as the antigen. Trp2 cellular uptake was improved when encapsulated by THMSN. The adjuvant-vaccine induced maturation of dendritic cells was confirmed with increased expression of costimulatory molecules such as CD86 and CD83 as well as high levels of proinflammatory cytokines in vitro. In vivo immunogenic activity tests resulted in mice immunized with Trp2@THMSN exhibiting the most Trp2-specific Th1 immune response. Sustained immunological memory was also exhibited in mice treated with Trp2@THMSN and subsequently undergoing a tumor rechallenge model.

**Figure 6:**
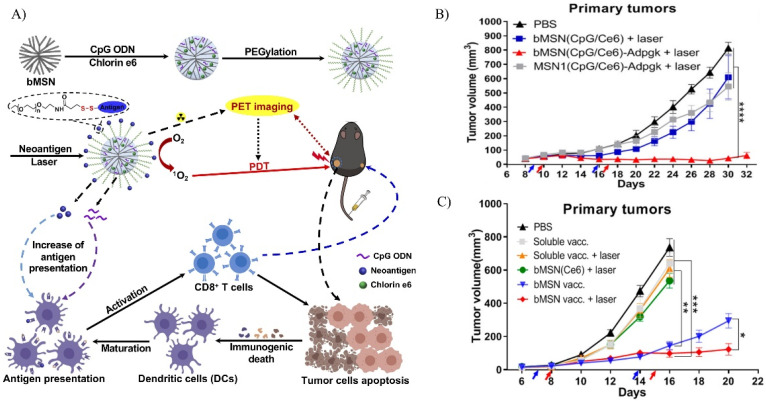
A biodegradable MSN as a platform for dual modality positron emission tomography-guided photodynamic therapy and immunotherapy. (**A**) A schematic of the fabrication of bMSN(CpG/Ce6)-neoantigen nanovaccines. The bMSN was synthesized using an oil-water biphasic reaction system. CpG and Ce6 were loaded into the bMSN using electrostatic and hydrophobic interactions, respectively. Neoantigen was added after PEGylation through disulfide bonds. (**B**) Antitumor study on MC-38 tumor-bearing mice. Tumor growth curve after treatment with each group is shown. (**C**) Antitumor study on B16F10 tumor-bearing mice. Tumor growth curve after treatment with each group is shown. Error bars, mean ± s.d., * *p* < 0.05, ** *p* < 0.01, *** *p* < 0.001, **** *p* < 0.0001. Reprinted (adapted) with permission from Ref. [125]. Copyright 2021, American Chemical Society.

A dual modality mesoporous silica DDS was investigated by Xu et al. The mesoporous silica combined photodynamic therapy (PDT) and immunotherapy onto one platform (Figure 6) [125]. The dendritic biodegradable MSN (bMSN) was synthesized using an oil-water biphase reaction and had a size around 80 nm. The bMSN was functionalized with APTES and PEG. CpG and photosensitizer chlorin e6 (Ce6) were co-loaded prior to PEGylation. bMSN co-loaded with CpG and Ce6 was found to induce the greatest amount of cytokine secretion, suggesting dendritic cell maturation. Combined therapy of vaccine and PDT in vivo on MC-38 colon carcinoma showed a significant improvement in antitumor efficacy over non-PDT vaccine (Figure 6B). Further evaluation of an immunosuppressive B16F10 melanoma model held up, with the combination therapy treatment resulting in a high survival rate with the lowest tumor growth (Figure 6C).

Seth et al. also developed a dual modality nanoparticle system for combined immunotherapy and photothermal therapy (PTT) [126]. A biocompatible and biodegradable polydopamine (PDA) core was used for photothermal properties and a mesoporous shell was coated on top. The immune stimulating agent gardiquimod, a toll-like receptor 7/8 (TLR 7/8) agonist, was loaded into the pores of the shell. The pores were subsequently capped with 1-tetradecanol, which is thermally activated at slightly above body temperature, allowing for controlled drug release when PDA heats up during PTT. In vivo tests of the therapeutic efficacy of the gardiquimod-loaded nanoparticle showed significant initial suppression of tumor growth. Adding PTT through NIR activation resulted in a significant increase in the survival rate of mice with B16-F10 melanoma model. The concurrent release of antigen from the cancer cells due to thermal ablation and adjuvant from the nanocarrier was necessary to achieve long-lasting immune response.

Duan and colleagues developed a pH-responsive MSN for the selective release of OVA antigen and CpG as immunostimulant/adjuvant [127]. A metal-organic framework (MOF) constituted by EuCl_3_ acts as the gatekeeper, preventing cargo from escaping the pores prior to delivery to the target site. Other gatekeepers such as supramolecular nanovalves, pH-sensitive linkers, and acid-degradable inorganics require complex preparation processes, so a MOF gatekeeper was utilized. The gated MSN can release cargo in the acidic tumor environment. The in vivo antitumor efficacy of the nanosystem was studied in B16-OVA-cell-bearing mice. The MSN-OVA@MOF@CpG treatment group resulted in the least tumor progression. The full treatment group had the highest concentration of CD8^+^ T cells in the tumor tissue compared to the other groups. Anti-metastasis efficacy of the full treatment group was also confirmed.

Another pH-responsive MSN was developed by Wagner et al. for the delivery of resiquimod (R848), a Toll-like receptor 7 and 8 agonist [128]. The gatekeeper used was a biotin-avidin complex with pH-responsive acetal linker to attach to the MSN. The biotin-avidin cap was chosen due to the strong noncovalent link between the two biocompatible molecules. The gatekeeper-modified MSN system was confirmed to have long-term stability at pH 7.0 and only opened at pH 5.5. Bone marrow-derived dendritic cells (BMDCs) were incubated with free R848, R848 loaded MSN, and pure MSN to evaluate activation of APCs. R848-loaded MSN and free R848 had similar levels of activation. The addition of OVA antigen to the system was studied. Although the APC activation using MSN-R848-OVA was lower than using MSN-R848, the CD8^+^ T cells proliferation was the highest in the MSN-R848-OVA group compared to MSN-R848 + free OVA and the other groups.

An Au-doped MSN designed by Ong et al. was loaded with CpG as a pathogen-associated molecular pattern (PAMP) and was covered with PEG-SH [129]. Extra-large pore MSN (XL-MSN) with pore sizes around 20–30 nm had Au nanoparticles attached to act as the photothermal therapy agent. The loaded CpG aids in inducing an immune response along with the antigens expelled from dead tumor cells post PTT. The PEG improves biocompatibility and decreases the cytotoxicity of the MSN. Assessing the in vitro immunostimulatory efficacy on BMDCs, Au@XL-MSN-CpG/PEG showed the highest expression level of CD11c and CD86 compared to free CpG and Au@XL-MSN/PEG. Cytokine concentrations were also the most elevated for Au@XL-MSN-CpG/PEG. The in vivo therapeutic efficacy was investigated, and Au@XL-MSN-CpG/PEG + NIR showed significant suppression of tumor growth, with a tumor volume approximately 1.5 times smaller than Au@XL-MSN-CpG/PEG without NIR. The Au@XL-MSN-CpG/PEG + NIR treatment group also showed the highest survival rate and had survivors after 37 days when all other groups died.

**Figure 7:**
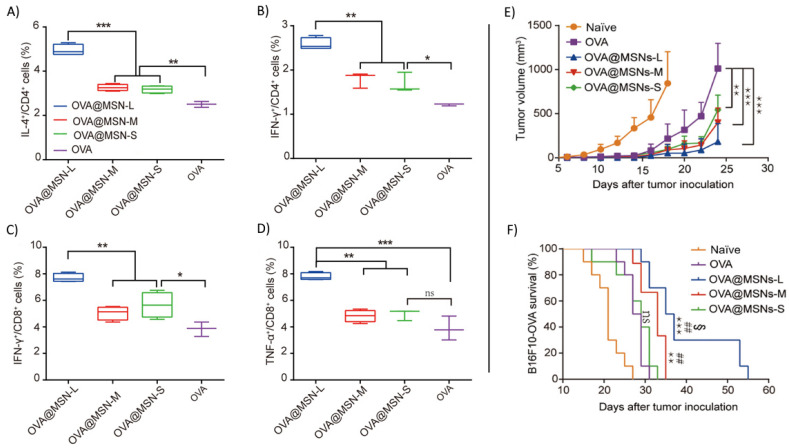
Small MSN of varying pore size loaded with OVA antigen. (**A**,**B**) Percentage of CD4^+^ T cells producing IL-4 and IFN-γ. (**C**,**D**) Percentage of CD8^+^ T cells producing IFN-γ and TNF-α. (**E**) Tumor growth curve of B16F10 cells. (**F**) Kaplan-Meier survival curve of mice comparing naïve, free OVA, and OVA@MSN. Error bars, mean ± s.d., * *p* < 0.05, ** *p* < 0.01, *** *p* < 0.001 versus free OVA; ## *p* < 0.01 versus OVA@MSNs-S; § *p* < 0.05 versus OVA@MSNs-M. Reprinted (adapted) with permission from Ref. [130]. Copyright 2021, Science.

Hong et al. investigated the effect of pore size on eliciting immune response (Figure 7) [130]. The MSN was designed to be approximately 80 nm to effectively drain to the lymph nodes and the pore sizes ranged from 7.8 nm as small (MSN-S), 10.3 nm as medium (MSN-M), and 12.9 nm as large (MSN-L). The different-pore-sized MSNs showed no difference in lymph node targeting and internalization in APCs but resulted in different levels of immune activation. MSN-L induced the highest level of interferon-γ (IFN-γ) and interleukin-4 (IL-4) secreting CD4^+^ T cells as well as IFN-γ and tumor necrosis factor–α (TNF-α) secreting CD8^+^ T cells (Figure 7A–D). Further assessment on antitumor efficacy revealed that MSN-L resulted in greater tumor growth suppression (Figure 7E) and had better survival time (Figure 7F). The larger pore size could possibly induce stronger cross-presentation of antigen. Larger pore size could also result in faster degradation time in the lymph nodes, leading to a stronger exposure of antigen to APCs.

A few other papers all reported on the use of mesoporous silica as a platform for enhancing cancer vaccine efficacy [131,132,133,134,135]. The collective data suggested that mesoporous silica improved cellular uptake of antigens by APCs such as dendritic cells and provided self-adjuvanticity. Additionally, combination therapy can be used to increase tumor suppression and allows for the simultaneous release of antigen and adjuvant. Therefore, mesoporous silica can be considered a versatile nanomaterial for use in cancer vaccine therapy.

### 5.3. Antibodies and Small Molecules

Antibodies and small molecule inhibitors can be considered similar to chemotherapeutics, and thus have comparable limitations such as nonspecific targeting and narrow therapeutic window due to expedited clearance. Nanomaterials such as mesoporous silica can ameliorate these issues and add other benefits such as higher loading capacity which allows for higher dosage.

Choi and colleagues encapsulated anti-programmed cell death ligand 1 antibodies (aPD-L1) in MSN and capped the system with ferumoxytol for a sequential magnetic resonance (MR) image guided system [136]. This DDS was employed after cabazitaxel chemotherapy for the treatment of prostate cancer. The ferumoxytol cap confers imaging capability and guidance to the target site using MR. The nanocarrier system showed a significant increase in the number of activated CD8^+^ T cells compared to free drug and showed a significant decrease in ratio of Treg cell, suggesting an adaptive immune response. This was further proven with a stable tumor size showing tumor suppression.

Zhao and coworkers encapsulated anti-programmed cell death protein 1 antibodies (aPD1) in MSN and applied a camouflage coating of cancer cell membrane [137]. The antibody aPD-1 inhibits the negative feedback pathway in the immune response by blockade of the PD-1/PD-L1 axis. The chemotherapeutic dacarbazine (DTIC), a drug used for the treatment of melanoma, was also encapsulated into the system. After DTIC kills cancer cells in the tumor mass, the released antigens could elicit an immune response alongside aPD-1. Assessment of in vivo efficacy showed that the fully loaded MSN treatment group had the highest rate of tumor inhibition and survival. In addition, in vivo administration of fully loaded MSN resulted in the highest concentration of secreted cytokines. This suggests not only chemotherapy efficacy but also immunotherapy efficacy from the aPD-1.

**Figure 8:**
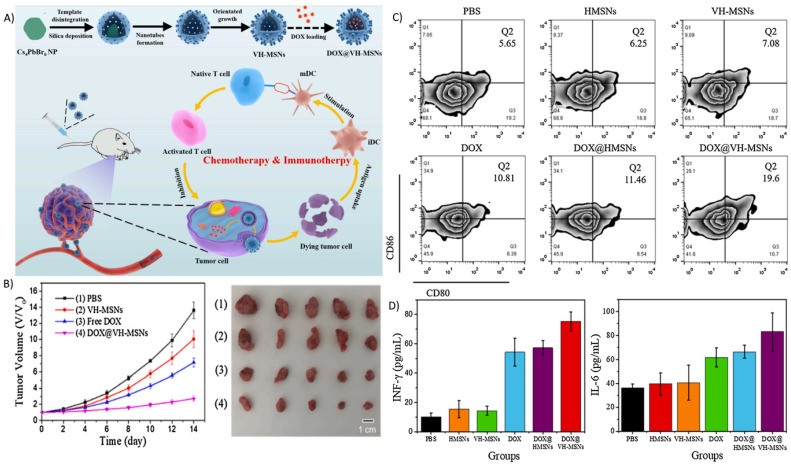
A virus-like hollow mesoporous silica nanoparticle was loaded with DOX. (**A**) Design scheme of the VH-MSN. (**B**) Tumor growth curve of 4T1 cells and the photograph of tumors collected posttreatment (**C**,**D**) Flow cytometry of the percentage of CD80^+^ and CD86^+^ matured APCs and the serum levels of cytokine IFN-γ and IL-6. Reprinted (adapted) with permission from Ref. [138]. Copyright 2021, Elsevier.

Xu et al. designed a virus-like HMSN (VH-MSN) loaded with DOX, a chemotherapeutic that also has immunogenic properties (Figure 8) [138]. A virus-like topographical modification enhances the internalization rate by tumor cells. It is possible that the virus-like spikes introduce a higher mechanical stress on the membrane, allowing for faster cellular uptake. In vitro cell viability assessment showed a significant difference in 4T1 cell viability between the free DOX and DOX@VH-MSN groups, with the DOX@VH-MSN group having around 2-fold lower cell viability at a DOX concentration of 50 µg/mL. Investigation of in vivo antitumor efficacy was conducted. It was found that there was greater inhibition of tumor growth in the DOX@VH-MSN treatment group than the free DOX group (Figure 8B). Assessment of the immunostimulatory properties of DOX was also conducted, with DOX treatment groups, notably the DOX@VH-MSN group, showing increases in CD80^+^ and CD86^+^ matured APCs. Additionally, cytokine IFN-γ and IL-6 levels were highest in the DOX@VH-MSN group (Figure 8C).

A thin shell HMSN (tHMS) loaded with DOX was designed by Li et al. and was co-administered with a cytotoxic T-lymphocyte associated protein 4 antibody (anti-CTLA-4) [139]. The anti-CTLA-4 can block inhibitory signals to T cells and therefore cause a more potent activation of T cells. Tumor immunogenicity was boosted by the effect of DOX and the adjuvant property of MSN. When comparing tHMS-DOX versus free DOX, tHMS-DOX had significantly higher cellular uptake of DOX and lower tumor cell viability in vitro. In vivo studies on antitumor efficacy were conducted with tHMS-DOX administered in one flank of the mouse and anti-CTLA-4 on the other flank. There was a significantly reduced tumor growth rate in the tHMS-DOX + anti-CTLA-4 group. Both flanks had inhibited tumor growth rate, indicating probable efficacy in metastatic cancer. Immune response was assessed based on the percentage of CTLA-4^+^ T cells, with the tHMS-DOX + anti-CTLA-4 group having the lowest overall percentage. Cytokine IFN-γ secretion in T cells was the highest in the tHMS-DOX + anti-CTLA-4 group, suggesting a complete immune response.

Delivery of cytokines to the tumor site can also induce immune response by activating APCs and T cells [148]. Recent works utilizing cytokines as immunostimulant have been performed by Kong et al. [140], Kienzle et al. [141], and Cheung et al. [142]. Through the use of mesoporous silica as a delivery platform, therapeutics such as antibodies, small molecule drugs, and cytokines all benefit from higher specificity.

## 6. Conclusions and Perspectives

Mesoporous silica as a nanomaterial has many favorable properties for drug delivery systems and nanoformulations. MSNs have tunable particle size, shape, pore structure, and pore size. Their surface functionalization is simple to execute, and their high surface area allows for high drug loading capabilities. Mesoporous silica also has moderate biocompatibility and can be modified to be more biocompatible [122,125]. MSN opens the possibility of multiple modalities of treatment and imaging in one versatile system. Lastly, scalable production of MSNs due to the relative ease and cost-effectiveness of fabrication allows for an easy translocation from laboratory to industry.

Considerations regarding safety should still be taken into account. Further study into the biocompatibility and biodistribution of mesoporous silica nanoparticles and their biodegradation byproducts should be conducted. Investigations on how to make MSNs safer in vivo, either through functionalization or modification of synthesis methods, are also crucial.

Application of mesoporous silica to cancer immunotherapy is still being developed, though with promising results. Mesoporous silica has self-adjuvanticity and can also co-load antigen and adjuvant together in a single platform. Then, the co-release or staggered release of the antigen and adjuvant can maximize the overlap of therapeutic windows. A very promising direction for MSN in immunotherapy is the design of multimodal cancer treatment. Dual modality cancer treatment using immunotherapy plus another therapeutic modality can synergistically enhance treatment efficacy. Using modalities such as chemotherapy, PDT, or PTT to kill a portion of cancer cells would release the tumor-associated antigens. These released antigens in conjunction with the immunotherapeutic would work to activate the immune response, mobilizing T cells against the cancer cells [126]. Although the effect of mesoporous silica on antigen presenting cells has been studied, there is a need to investigate the interactions between MSN and other innate immune cells. Afterwards, MSN can be finetuned to improve the immunological response of those cells. As for cancer vaccines, more investigations into patient-specific tumor-associated antigens rather than simple model antigens are needed.

Considerable progress has been made in the application of mesoporous silica as a versatile nanomaterial in multiple fields over the past few decades. Immunotherapy is one of the more recent applications of mesoporous silica in the field of cancer therapy. Extensive effort is needed to translate the MSN-based nanoformulations into working products for clinical use. Further research and investigation to improve material properties and resolve issues and concerns of mesoporous silica as a nanomaterial will expedite the process.

## Figures and Tables

**Table 1:** Types of ordered mesoporous silica.

Type	Pore Size (nm)	S_BET_^1^ (m^2^/g)	Structure	References
MCM-41	1.5–8	1000–1200	2D hexagonal P6 mm	[31,39,40]
MCM-48	2–5	1000–1250	3D cubic Ia3d	[37,39,40]
SBA-15	4–10	700–1000	2D hexagonal P6 mm	[38,40,41]

^1^ Surface area based on Brunauer–Emmett–Teller (BET) theory.

**Table 2:** Targeting modality for MSNs.

Molecule Family	Molecule Type	Method of Action	References
Protein	mAbs	Specific binding to tumor cell surface antigens	[89,90,91,92]
	Fabs	Specific binding to tumor cell surface antigens	[93,94]
Peptide	RGD	Binding to the overexpressed integrin α_V_β_3_	[95,96]
	CPPs	Interactions with cell membrane or surface proteins	[97,98,99]
Nucleic Acid	Aptamers	Specific binding to overexpressed receptor on tumor cell surface	[100,101,102]
Small Molecule	Folate	Targets the overexpressed folate receptor α	[75,95,103]
	Hyaluronic Acid	Targets the overexpressed CD44	[96,104]

**Table 3:** Summary of recent works on mesoporous silica as a platform for cancer immunotherapy.

Mesoporous Silica Type	Property	Payloads	References
bMSN	50–60 nm, biodegradable	Carbon Nanodot	[121]
MSN	432 ± 198 nm, acetalated dextran coating, spermine	Cancer cell membrane, Trp2	[122]
XL-MSN	100–200 nm, pore size ~25 nm	OVA, CpG	[123]
THMSN	~200 nm, pore size 3.6 nm, thickness ~22 nm, PEI coating	Trp2	[124]
bMSN	~80 nm, pore size 5–10 nm, PEGylated, biodegradable	CpG, Ce6	[125]
HMSN	340 ± 40 nm, thickness ~80 nm, 1-tetradecanol capped	PDA core, Gardiquimod	[126]
MSN	~100 nm, MOF capped	OVA, CpG	[127]
MSN	180–280 nm, pore size 3.2 nm biotin-avidin capped	Resiquimod	[128]
XL-MSN	~130 nm, pore size 20–30 nm, PEGylated	AuNP, CpG	[129]
MSN	~80 nm, pore size 7.8 nm/10.3 nm/12.9 nm	OVA	[130]
DMON	~200 nm, PEI coating, biodegradable	OVA, CpG	[131]
bMSN	~80 nm, pore size 5–10 nm, biodegradable	CDA	[132]
MSN	46.6 ± 0.3 nm, pore size 2.3 nm, PEGylated, TA-silane functionalized	cdG, RITC	[133]
XL-MSN	~100 nm	β-NaYF4:20%Yb,2%Er upconversion nanoparticles, MC540, Tumor cell fragment	[134]
MSN and MSR	Particles: ~150 nm, pore size 20–30 nm Rods: length 86 µm, width 14.5 µm	OVA, CpG, GM-CSF	[135]
XL-MSN	~300 nm, pore size 14.6–25 nm, ferumoxytol capped	aPD-L1, ferumoxytol	[136]
MSN	151.78 ± 5.57 nm, cancer cell membrane coated	Dacarbazine	[137]
VH-MSN	~260 nm, virus-like topography	DOX	[138]
tHMS	~200 nm, thickness ~20 nm	DOX	[139]
bHMSN	~180 nm, lipid bilayer coated, biodegradable	DOX, ATRA, IL-2	[140]
DMSN	120–205 nm, pore size 6.7–12.7 nm, PEI-PEG capped/coated	TNF-α	[141]
MSR	length 70 µm, width 4.5 µm, pore size 10.9 nm	aCD3, aCD28, IL-2	[142]

## Data Availability

Not applicable.
