# Correction: Yu et al. Recent Advances of Mesoporous Silica as a Platform for Cancer Immunotherapy. *Biosensors* 2022, *12*, 109

**DOI:** 10.3390/bios12100794

**Published:** 2022-09-27

**Authors:** Albert Yu, Xiaoyong Dai, Zixian Wang, Huaqing Chen, Bing Guo, Laiqiang Huang

**Affiliations:** 1 Precision Medicine and Healthcare Research Center, Center for Biotechnology and Biomedicine, Shenzhen Key Laboratory of Gene and Antibody Therapy, State Key Laboratory of Chemical Oncogenomics, State Key Laboratory of Health Sciences and Technology, Tsinghua-Berkeley Shenzhen Institute (TBSI), Institute of Biopharmaceutical and Health Engineering, Shenzhen International Graduate School, Tsinghua University, Shenzhen 518055, China; 2 School of Science and Shenzhen Key Laboratory of Flexible Printed Electronics Technology, Harbin Institute of Technology, Shenzhen 518055, China

## Additional Affiliation(s)

In the published publication [1], there was an error regarding the affiliation(s) for **Albert Yu, Xiaoyong Dai, Zixian Wang, Huaqing Chen, and Laiqiang Huang**. In addition to affiliations **1, 2 and 3**, the updated affiliations should include: **1. Precision Medicine and Healthcare Research Center, Center for Biotechnology and Biomedicine, Shenzhen Key Laboratory of Gene and Antibody Therapy, State Key Laboratory of Chemical Oncogenomics, State Key Laboratory of Health Sciences and Technology, Tsinghua-Berkeley Shenzhen Institute (TBSI), Institute of Biopharmaceutical and Health Engineering, Shenzhen International Graduate School, Tsinghua University, Shenzhen 518055, China. 2. School of Science and Shenzhen Key Laboratory of Flexible Printed Electronics Technology, Harbin Institute of Technology, Shenzhen 518055, China.** The authors state that the scientific conclusions are unaffected. This correction was approved by the Academic Editor. The original publication has also been updated.
